# The effect of ethical leadership on subjective wellbeing, given the moderator job satisfaction (a case study of private hospitals in Mashhad)

**DOI:** 10.1186/s12912-020-00496-w

**Published:** 2020-11-30

**Authors:** Azar Kaffashpoor, Samaneh Sadeghian

**Affiliations:** 1 Management Department, Faculty of Economics and Administrative Sciences, Ferdowsi University of Mashhad, Mashhad, Iran; 2 Department of Management, Tabaran Institute of Higher Education, Mashhad, Iran

**Keywords:** Ethical leadership, Job satisfaction, Organizational performance, Subjective wellbeing (SWB)

## Abstract

**Background:**

The emerging ethical leadership, a unique approach in leadership viewpoint, has provided the ground for constructing and advancing individual and managerial efficiency by highlighting ethics in organizations. The present study aims to investigate the influence of Ethical Leadership on Subjective Wellbeing, Given the Moderator Job Satisfaction in Private Hospitals in Mashhad.

**Methods:**

This descriptive-correlational research design stud was conducted in 2015–2016 to inspect the possible effect of ethical leadership on subjective wellbeing and job satisfaction, as dependent and mediator variables, among the Iranian private hospitals’ nurses in Mashhad. Simple random sampling method was used to select the sample of 166 nurses out of the population of 730 nurses, in total. The valid and reliable adapted version of the questionnaire designed by Yang (2014) was used to collect the data, and structural equation modeling (SEM) was used to analyze the data set.

**Results:**

The results showed that there is a positive significant correlation between ethical leadership and job satisfaction. More specifically, the findings indicated that Ethical leadership affected the subjective wellbeing of nurses through job satisfaction both directly and indirectly.

**Conclusions:**

The findings illustrated that focus on ethics and ethically-oriented leaders in hospitals, enriched by job satisfaction can lead to the nurses’ subjective wellbeing by providing them a positive climate.

## Background

The issue of organizational health, fulfilled through the law or ethics, is a vital consideration in any organization to survive and long-term success. Thus, ethics and morality must be taken into account as key sources of ethical guidelines for the leaders and personnel in organizations. Besides, very recently, advancing in issues such as Altruism, honesty, empowerment, fairness and justice (e.g., [1, 2]) has been of much interest to the researchers because of current scandals, which accordingly led to the investigation of ethical leadership concept in the early twentieth century [3].

Consistent with the social learning theory, personnel acquire the way of communicating with others through imitating and monitoring organizations’ leaders’ [4]. However, the number of studies concentrating on the practical application of this theory, specifically about ethical leadership, is so scarce [5]. From a social learning viewpoint, ethical leaders are considered as role models forming suitable manners and standards in an organization [6]. Thus, ethical leadership is defined as the confirmation and advancement of normatively suitable personal and interpersonal behavior in two-way communications, reinforcement and decision-making [6–9]. This leadership model can be perceived in more tangible terms by a triple behavioral dimension: (1) leader’s ‘fairness’ which refers to being fair, trustworthy and honest meaning that ethical leaders treat others with respect, do not differentiate among others and make fair decisions. (2) leader’s ‘power sharing’ behavior which refers to giving juniors a voice, listen to their input, and allow them to take part in decision-making. (3) leader’s ‘role clarification’ referring to working clearly, clarifying expectations, and communicating openly in order to let the followers understand what is expected from them [10]. According to the theory of social learning, followers have a tendency to pay attention and follow their ethical leaders’ outlooks, values, and manners because leaders’ attraction and trustworthiness as role models and source of guidance draw attention to their modeled behavior [6]. Some probable positive effects of ethical leadership are job satisfaction (e.g., [11–14]), and employee wellbeing (e.g., [14, 15]).

Subjective wellbeing, in working and managerial settings, refers to the employee’s insight and assessment of the quality of (working) life, social and psychological working in those settings [16]. Previous studies confirmed the relation between leader’s manners and employee’s wellbeing (e.g., [17], (10.1097/JOM.0b013e31817e918d)]), and the effect of leadership styles such as transformational, honorable and ethical leadership on the employee’s subjective wellbeing (e.g., [15, 18, 19]). As the Conservation of Resources (COR) theory proposes, people struggle to remember, protect, and construct resources with the fear of the potential or actual loss of these valued resources. Resources, then, are the single essential unit for understanding stress. COR theory asserts that resources, such as ethical leadership, aid employees to gain more resources. This starts a positive spiral of resources, which can positively impact on wellbeing. Ethical leaders can offer job resources by effectively protecting employees, defending them from injustice or mobilizing job resources, which positively affect employee’s wellbeing [20]. Ethical leaders are caring, honest and reliable. They encourage employees to state their worries and make fair decisions on importance issues [21]. In doing so, ethical leaders are impartial and truthful and provide personnel with a safety environment to fall back on while experiencing low levels of wellbeing at work settings. Accordingly, personnel receive help, attention, and emotional care from their leader. Accordingly, Zou et al., [22] show the effect of spiritual leadership on the Chinese nurses’ subjective well-being [23]. Teimouri et al., [24] also confirmed the effect of ethical leadership on psychological wellbeing of employees [25]. Sarwar et al., [26] also show the direct positive relation between ethical leadership and employee well-being [22]. Thus, according to the previous research, the following hypothesis is proposed:

Hypothesis 1 – The leader’s ethical leadership has a significant effect on the subjective wellbeing of nurses.

Job satisfaction is a multidimensional notion related to a range of psychological and social elements [24]. Moreover, previous studies found a significant association between ethical leadership style and different organizational successes like job satisfaction [24, 26]. Tu et al., [14] confirmed the effect of supervisors’ ethical leadership on employee job satisfaction [13]. According to Robbins and Coulter [27], job satisfaction is an employee’s general attitude to his/her job [28]. According to Qing et al., (2018), ethical leadership can predict job satisfaction positively in public sector organizations [10]. Moreover, Freire and Bettencourt [13] showed that ethical leadership had a positive effect on nurses’ job satisfaction. Ruiz-Palomino et al. [29] stated that the significant connection between ethical leadership and job satisfaction was because of the central role of leaders in forming organizational culture and setting [30]. Kim and Brymer [8] also stated leaders’ ethical conducts have positive relationships with an employee’s enhanced job satisfaction with the current working condition and amount of payment [8]. Thus, based on the previous research, the following hypothesis is proposed:

Hypothesis 2 – The Leader’s ethical leadership has a significant effect on the job satisfaction of nurses.

The mediation effect of job satisfaction on the relationship between ethical leadership and subjective wellbeing has not been sufficiently addressed in literature. Among rare existent studies, Ahanchian et al., [31] shows the positive effect of ethical leadership on life well-being of nurses mediated by job Satisfaction. However, they did not consider the subjective wellbeing of employees [27]. Mehari [32] also shows that job satisfaction indirectly mediates the effect of transformational leadership and employee well-being [29]. Indeed, it is the leader’s ethical behavior that either directly or indirectly influences followers’ subjective well-being. Hence, in line with these previous research findings, the following hypothesis is suggested:

Hypothesis 3 –Job satisfaction has a significant effect on the subjective wellbeing of nurses.

Hypothesis 4 – Job satisfaction significantly mediates the effect of ethical leadership on the subjective wellbeing of nurses.

**Fig. 1:**
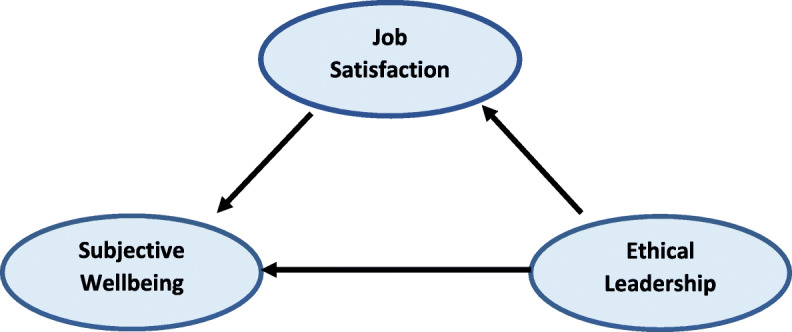
Research Conceptual model (Source: Yang, 2014)

Given the above three main hypotheses, the research conceptual model is presented as follows (Fig. 1):

## Methods

The present applied research was conducted as a descriptive-correlational survey in which the data were analyzed through covariance matrix using structural equation modeling (SEM) technique. The nurses of private hospitals in Mashhad (i.e. Bent-ol-Hoda, Mehr, Sina and Razavi hospitals) who were a sum of 730 in number comprised the statistical population of the present study.

As the statistical population was from several hospitals, the stratified sampling method has been used. First, four hospitals of Bent-ol-Hoda, Mehr, Sina and Razavi hospitals were randomly selected from the list of Mashhad private hospitals. By considering the error level for 5% in Cochran formula, the sample size was accurately estimated at 95% confidence interval and significance level of 5%, giving a sample size of 157 persons. Then, random sampling was performed from each hospital according to the ratio of sample to community (equivalent to 0.23). However, anticipating that a certain number of the questionnaires may not return, 200 questionnaires were actually distributed among nurses. Of these questionnaires, 166 completed questionnaires were eventually used in the analysis phase. The size of the population and the sample by stratums are given in Table 1.

**Table 1:** Population and sample size

Hospital	No. Nurses	Sample size
Bent-ol-Hoda Hospital	221	166/221*221 = 50.25
Mehr Hospital	148	34
Sina Hospital	107	24
Razavi hospitals	254	58
Sum	730	166

Due to the limited number of populations, Cochran formula for finite population was used to determine the sample size. Using this sampling method, 157 participants were chosen (t 95% confidence interval and significance level of 5%) after pretesting a preliminary sample of 30 questionnaires, and substituting the Cochran formula’s 5% error level. However, from the total number of 200 distributed questionnaires, only 166 ones were returned and used in the study’s analysis step.

To assess the variables, 17 standard measures of Yang [33, 31] were used. Translation – back – translation method was used to make the measures ready to apply in the Iranian context. Five experts in the field of management and five working nurses in Mashhad’s private hospitals verified the face and content validity of the final version of the questionnaire including 4 Job satisfaction’ items, 9 ethical leadership’s items, and 4 subjective wellbeing’ items. All the items were rated on a five-point Likert scale ranging from strongly disagree (1) to strongly agree (5). In addition, the questionnaire’s construct validity and reliability (internal consistency) were checked and confirmed using a confirmatory factor analysis and Cronbach’s alpha, respectively. The result of calculated alpha showed an acceptable value of .81 which approves its sufficient reliability. Table 1 illustrated the calculated Cronbach’s alphas for each variable, and Table 2 presents the results of the Pearson correlation test. For data analysis, Pearson correlation coefficient and SEM were used to estimate zero order correlation coefficients and to test the goodness of fit in structural equations using SPSS24 and Smart PLS3.

**Table 2:** Mean test

Variable	Mean	St. Dev.	Sig	Status
(1) Ethical leadership	3.97	.52	0.000	Appropriate
(2) Job satisfaction	3.33	.69	0.000	Appropriate
(3) SWB	3.25	.68	0.000	Appropriate

## Results

### Descriptive statistics

Respondents’ demographic information was analyzed in terms of 4 variables: gender, age, education and years of service (work experience). According to the descriptive analysis, 68.1% of respondents were women and 31.9 were men; 32.5% aged between 20 and 30, 38.6% between 31 and 40, 22.9% between 41 and 50, 4.8% between 51 and 60, and 1.2% above 61; 15.7% were high school graduates, 18.7 junior college graduates, 42.8% university undergraduates, 12.7% university graduates, and 10.2% Ph.D. graduates; 28.9% had less than 5 years of working experience, 21.7% between 5 and 10 years, 21.1% between 10 and 15 years, 12% between 15 and 20 years, 12.7% between 20 and 25 years, and 3.6% worked more than 25 years.

Table 2 presents Cronbach’s alpha, mean response and the respective standard deviation of each variable. Note that the Cronbach’s alpha for job satisfaction was initially .42, but after the exclusion of one item it rose to .82.

As it can be observed in the above table, the mean responses for all variables are in an appropriate mean, among which the highest amount belongs to ethical leadership.

As the presence of a pairwise linear correlation between variables is a necessary assumption in applying the latent variables method in structural equation modeling (SEM), first, for each pair of variables, Pearson correlation test was run and the result is presented in Table 3.

**Table 3:** The results of Pearson correlation test

Variable	(1)	(2)	(3)
(1) Ethical leadership	–	.184*	.103
(2) Job satisfaction	.184*	–	.560**
(3) SWB	.103	.560**	–

** correlation at significance level of *p* < 0.01; * correlation at significance level of *p* < 0.05

As Table 2 shows, the strongest correlation is that of job satisfaction and SWB (*r* = 0.560), and the smallest correlation exists between SWB and ethical leadership (*r* = 0.103). In addition, all the estimated paired correlation coefficients are positive and significant.

### Validity and reliability of measurement and structural model

The research model was analyzed by Smart PLS 3 employing structural equation modeling (SEM). The validity and reliability of the constructs was estimated using factor loadings, Cronbach’s Alpha, composite Reliability average variance extracted (AVE) shown in Table 4.

**Table 4:** Validity and reliability test

Construct	Items	Factor loadings	Cronbach’s alpha	Composite reliability	Average variance extracted (AVE)
Ethical Leadership	1	0.718	0/884	0/907	0/523
	2	0.624			
	3	0.807			
	4	0.799			
	5	0.816			
	6	0.722			
	7	0.670			
	8	0.589			
	9	0.718			
Job Satisfaction	10	0.848	0/768	0/865	0/683
	11	0.891			
	12	0.733			
Subjective Wellbeing	13	0.734	0/790	0/856	0/543
	14	0.731			
	15	0.763			
	16	0.764			
	17	0.689			

As shown in Table 4, all factor loadings were more than 0.5, shown appropriate reliability. Cronbach’s α incidents were above 0.7 value showing satisfactory reliability. Moreover, the value of composite reliability and AVE were more than 0.7 and 0.5 respectively, showing satisfactory reliability [32].

To test the hypotheses, the partial least squares structural equation modeling (PLS-SEM) by Smart PLS 3 was employed. To test the fitness of structural model, R2 and Q2 measures were shown in Table 5.

**Table 5:** Goodness of fit of structural model

	SSO	SSE	Q^2^ (=1-SSE/SSO)	R Square
Ethical Leadership	1494/000	1494/000		
Job Satisfaction	498/000	432/517	0/131	0/213
Subjective Wellbeing	830/000	626/835	0/245	0/495

As shown in Table 5, The first criterion for examining the structural model is the coefficient of determination R2 related to the endogenous (dependent) latent variables in the model and shows the effect of an exogenous variable on an endogenous variable. The strength of this effect interpreted with three values of 0.19, 0.33 and 0.67 as weak, medium and strong values [32]. Accordingly, the result shows that the model can predict 0.213% of job satisfaction changes, measured as a mediate effect. Moreover, 49% of subjective wellbeing changes predicted by the model, showed strong effects of exogenous variables of the model on subjective well-being. Q2 value determines the strength of the model in predicting dependent variables. Hair et al., [34] considered three values of 0.02, 0.15 and 0.35 as low, medium and strong predictive strength [32]. As shown in Table 4, the value of Q2 for all dependent variables were moderate.

### Test of hypotheses

**Fig. 2:**
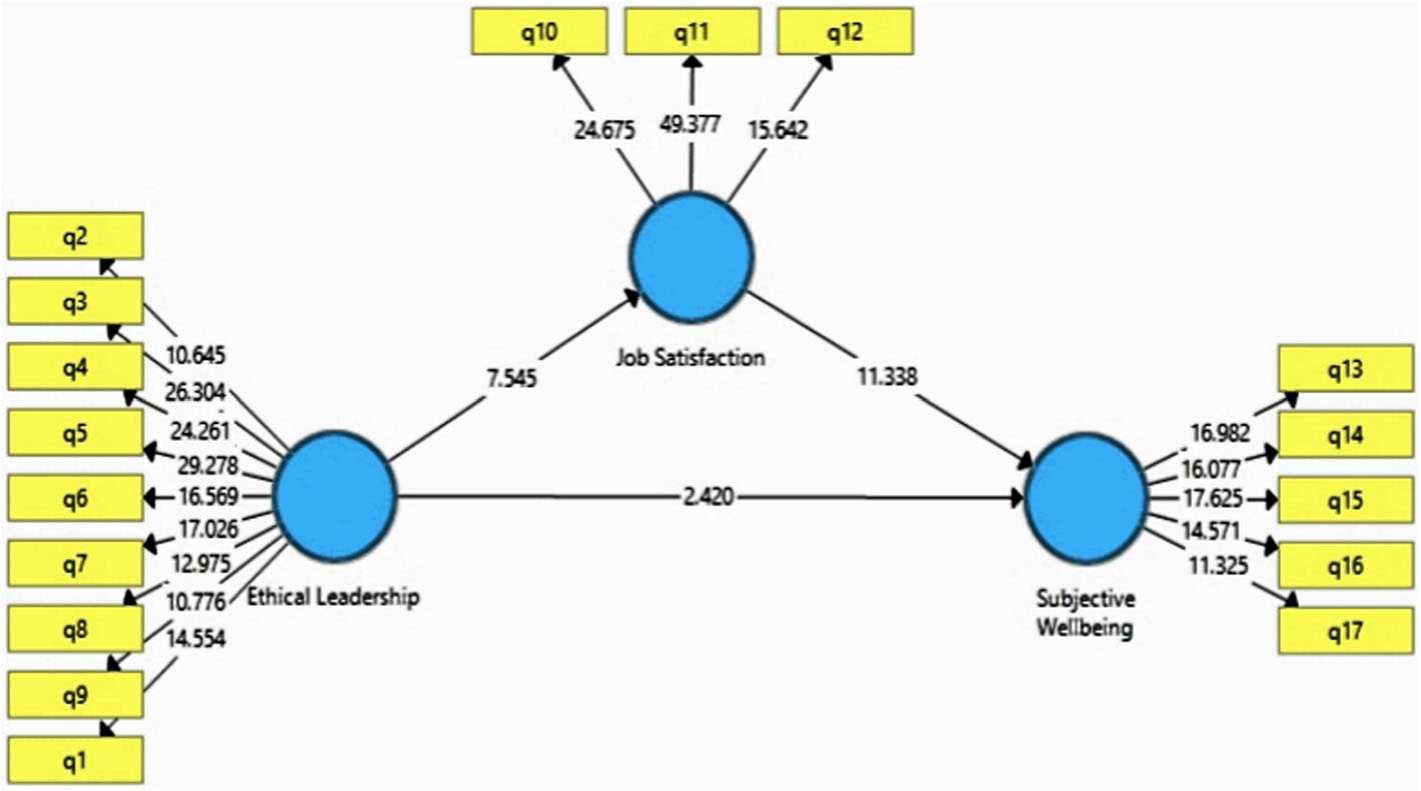
SME model in *T*-value mood

To test the hypotheses, PLS-SEM by Smart PLS 3 was employed. Figure 2 shows the SME model in *T*-value mood:

According to Fig. 2, the relationship between variables are significant when the *T*-value was more than 1.96. As shown in Fig. 2, all hypotheses of the research were accepted. The result of hypothesis tests is shown in Table 6.

**Table 6:** Summary of hypotheses testing results

	Standard path coefficients	*T*-value	Results
Ethical Leadership - > Job Satisfaction	0.462	7.445	Supported
Job Satisfaction - > Subjective Wellbeing	0.619	11.338	Supported
Ethical Leadership - > Subjective wellbeing	0.155	2.420	Supported

According to Table 4, all hypothesis was supported. Thus, the impact of ethical leadership (β = 0.155, *T*-value = 2.420) and job satisfaction (β = 0.619 *T*-value = 11.338) on subjective wellbeing are significant. Moreover, the effect of ethical leadership on job satisfaction is supported (β = 0.462 *T*-value = 7.445).

### Testing mediation effects

To measure mediation effect of job satisfaction in the relationship between ethical leadership and subjective wellbeing, the indirect effect shown in Table 7.

**Table 7:** Summary of hypotheses testing results

	Indirect effect		Total effect	
	Standard path coefficients	*T* Value	Standard path coefficients	*T* Value
Ethical Leadership - > Job Satisfaction - > Subjective Wellbeing	0.286	7.160	0.443	6.705

As shown in Table 4, the indirect effect of ethical leadership on subjective wellbeing through job satisfaction is confirmed (β = 0.286, *T*-value = 7.160), shown the mediation effect of job satisfaction. The result also shows that the indirect effect of ethical leadership on subjective wellbeing through job satisfaction is more than the direct effect of ethical leadership on subjective wellbeing (β = 0.155, *T*-value = 2.420). Accordingly, the total effect (direct effect* indirect effect) of ethical leadership on subjective wellbeing is 0.443.

## Discussion

The aim of this study was to investigate the relationship between moral leadership and subjective wellbeing with the mediating role of job satisfaction among nurses in four private hospitals in Mashhad. This study is based on social identity theory which states that nurses’ behavior is influenced by the behavior of their managers and leaders, leading nurses to be identified in the workplace. The results of data analysis showed that there is a significant relationship between research variables. In general, this study had two main conclusions: The first one reveals that ethical leadership directly and indirectly affects nurses’ happiness. Therefore, we can conclude that moral leadership affects not only the work of an individual but also the subjective wellbeing of nurses, which is in line with Yang [31], Teimouri et al., [25] and Sarwar et al., [22]. Zou et al., [22] also show the effect of spiritual leadership on the nurse’s subjective well-being [23]. In this regard, the authors also conducted interviews with the nurses under study, which they also confirmed and stated that the ethical leadership style in the hospital can greatly affect their mental happiness and feeling of happiness. The second conclusion indicates that ethical leadership affects nurses’ job satisfaction, which is in line with the study of Ngabonzima et al., [35], Ganji et al., [36] and Tu et al., [15]. Moreover, the results also are in line with Freire and Bettencourt [12] study, shown the positive effect of ethical leadership on nurses’ job satisfaction. In this regard, a number of additional interviews were conducted with some nurses who also emphasized that ethical leadership style has an impact on their job satisfaction. The results also confirmed the mediation effect of job satisfaction on subjective wellbeing, which is in consistent with Ahanchian et al. [27] and Mehari [29] studies.

These results, seen in a broader context, point to the increasing importance of ethically-oriented leadership in the target organization and healthcare institutions. Appreciating admirable human values, and being supportive and inspiring to nurses and other employees in the healthcare sector would create a feeling of usefulness and efficacy, pave the way for effective task performance and satisfy psychological needs. The ethically oriented approach in leadership has many benefits for organizations, which are more obvious and noticeable in the health management system and the nursing staff due to the dominance of an intimate and strongly emotional atmosphere. The particular working state and atmosphere of hospitals require such closeness and company among nurses, staff members, physicians, and patients. Naturally, through this closeness, many ethical issues are highlighted. The prevalence of ethical leadership in hospitals makes managers of sections and units further committed to ethical principles and somehow set an example for subordinates. We may then expect practice of ethical behaviors from nurses and staff members. Leadership, by definition, means influence and persuasion of others as distinguished from management by its emphasis on voluntary compliance of subordinates. Indeed, leadership behavior, in itself, is effective, supportive, stimulating and significant and when seasoned by the consideration of ethics and as an ethical model, it takes a more pleasing color which significantly contributes to the establishment of ethical rules in the organization. Our results, in general, support the promotion of ethical leadership in private hospitals through formal planning and top management initiatives both for the good of employee’s working conditions and the quality of the services provided.

## Conclusion

In the present study, the correlation between ethical leadership and job satisfaction, and also its (their) relation with subjective wellbeing with and without the nursing staff’s job satisfaction as the mediator was examined in Mashhad. The results indicated that ethical leadership has a significant influence on job satisfaction and subjective wellbeing. Accordingly, results show that job satisfaction, among nurses, was facilitated by the influence of ethical leadership on subjective wellbeing. Accordingly, because the principled leader of the hospital increases the feeling of satisfaction in nurses by adhering to ethical principles, fair and equitable resolution of issues, listening to the conversations and concerns of nurses, and sympathy and solving their problems, then nurse job satisfaction and subjective well-being would be increased. Ethical leadership and the basic standards of the business as well as establishing, validating and collaborating these values and standards must be integrated into the central standards and ideas of the firm by the hospital’s directors and managers. Moreover, administrators and chief managements should actively be engaged in practicing the ethical leadership and should also show a good ethical management skill.

## Data Availability

The datasets used and/or analyzed during the current study are available from the corresponding author on reasonable request. For having this data, please contact AK. kafashpor@um.ac.ir
